# Efficacy of tiapride in the treatment of alcohol abstinence, a review of the literature

**DOI:** 10.1192/j.eurpsy.2026.10803

**Published:** 2026-07-31

**Authors:** G. F. Varandas Rodrigues, A. F. Teixeira

**Affiliations:** 1 Psychiatry and Mental Health, Unidade Local de Saúde do Alto Minho, Viana do Castelo; 2 Centro de Respostas Integradas de Braga, Guimarães, Portugal

## Abstract

**Introduction:**

Alcohol use disorder (AUD) is a prevalent clinical condition, often associated with a high psychosocial impact and significant morbidity. The pharmacological approach to this condition aims to control the symptoms of alcohol withdrawal syndrome (AWS), reduce craving and prevent relapses.Tiapride is an atypical antipsychotic that has been used by health professionals as an adjuvant therapeutic option in this context, given its mechanism of action at the level of the central dopaminergic system and its favorable clinical safety profile.

**Objectives:**

Review the literature regarding of scientific studies on the efficacy, tolerability, and maintenance of alcohol abstinence with the use of tiapride in individuals with AUD.

**Methods:**

Non-systematic review based on a search for scientific articles from the last 25 years, in English or Portuguese, available in Pubmed with the MeSH terms: ‘tiapride’ and ‘alcohol withdrawal syndrome’.

**Results:**

Five clinical studies evaluating the efficacy of tiapride in the treatment of AUD were included. Most of the trials were carried out in hospital admission, with comparisons with placebo, benzodiazepines, carbamazepine or pregabalin. Amongst the studies included, the systematic review by Zangani et al. (2022) stands out, which identified six randomised clinical trials in which tiapride demonstrated efficacy in reducing craving and behavioural symptoms during abstinence. However, the studies included were from the 1980s and 1990s, with small samples and short follow-up periods. On the other hand, in the Cochrane review (2018), no statistically significant differences were identified between tiapride and placebo in terms of the relevant clinical evolution, the dropout rate or the patients’ quality of life, thus not allowing for reliable conclusions about the efficacy of tiapride in AUD. The article by the Italian Society of Alcoholology (2019) concluded that tiapride can be useful in the treatment of AWS, especially in patients with contraindications to benzodiazepines, and suggested its use in association with anticonvulsants in severe cases and/or with psychotic symptoms.

**Conclusions:**

Tiapride is a substituted benzamide, a selective antagonist of D2/D3 dopamine receptors, with a special affinity for the dopaminergic pathways of the limbic system and striatum, which gives it a useful pharmacological profile for controlling symptoms such as psychomotor agitation, anxiety and craving. Since there is no significant hepatic metabolism, it is also a safe option for patients with mild to moderate liver disorders. Some studies suggest that tiapride may have a beneficial effect in reducing anxiety symptoms and craving during alcohol withdrawal, but have not shown superiority over placebo or other commonly used drugs. However, it could be a pharmacological alternative for patients with contraindications to the use of benzodiazepines or with neuropsychiatric comorbidities.

**Disclosure of Interest:**

None Declared

